# Supplementary figures and images for: Exploring the potential of deep-blue autofluorescence for monitoring amyloid fibril formation and dissociation
Source: PeerJ. 2019 Aug 16;7:e7554. doi: 10.7717/peerj.7554 (PMC6699583; doi:10.7717/peerj.7554)

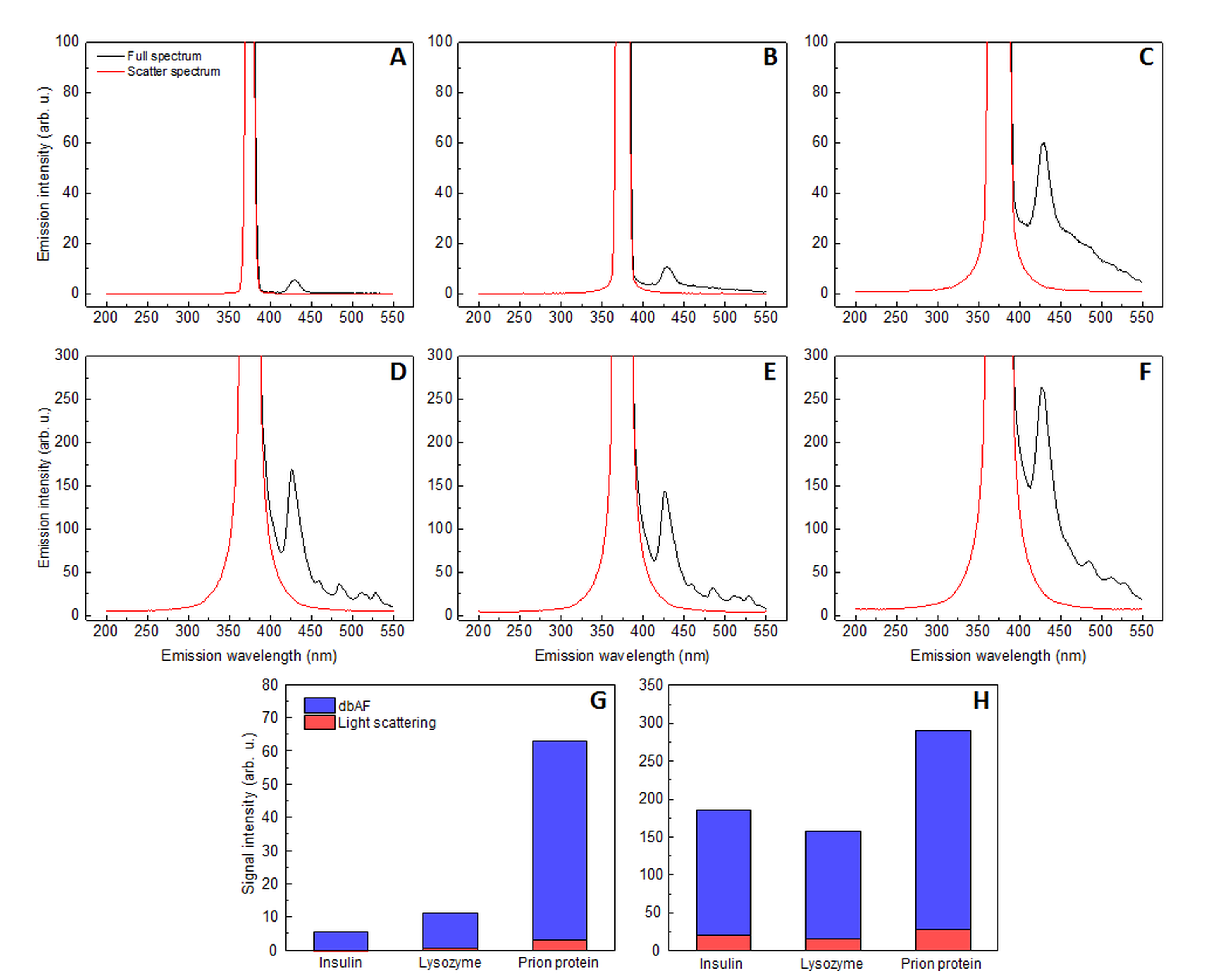

Supplement: Figure S1 — Light scattering and full spectrum comparison at initial insulin (A), lysozyme (B) and prion protein (C) seeded growth conditions, as well as at the end of the reaction when insulin (D), lysozyme (E) and prion protein (F) fibrils are formed. The light scattering spectrum which would be present without dbAF was determined by superimposing a mirror image of light scattering intensity from the excitation wavelength (375 nm) to the shorter wavelength region onto the region from the excitation wavelength to the longer wavelength region. Effect of light scattering at the measured wavelength (428 nm) on the total signal intensity at initial seeded growth conditions (G) and at the end of each reaction (H). Excitation and emission slits were used as in the aggregation experiments. [file peerj-07-7554-s001.png]

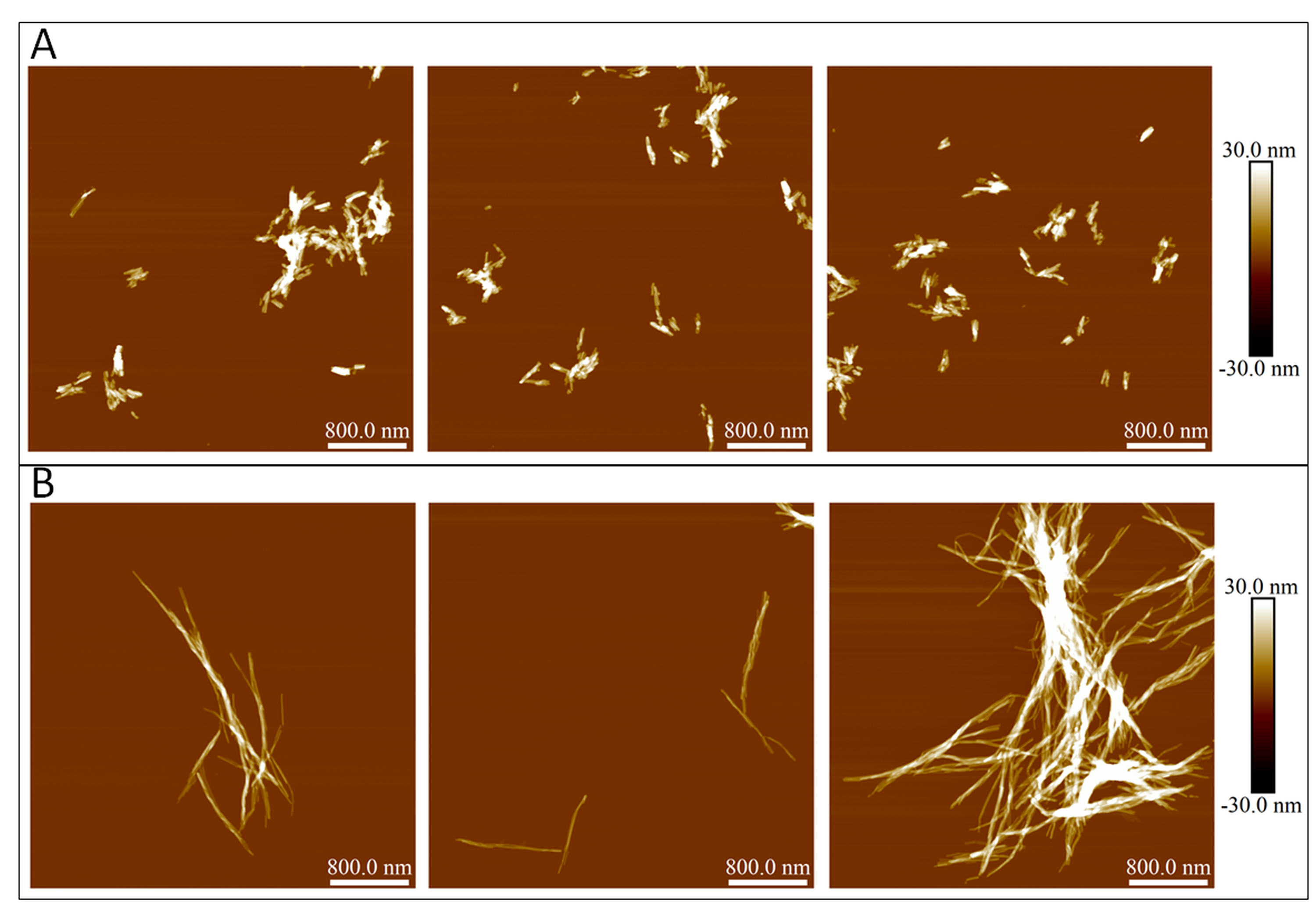

Supplement: Figure S2 [file peerj-07-7554-s002.png]

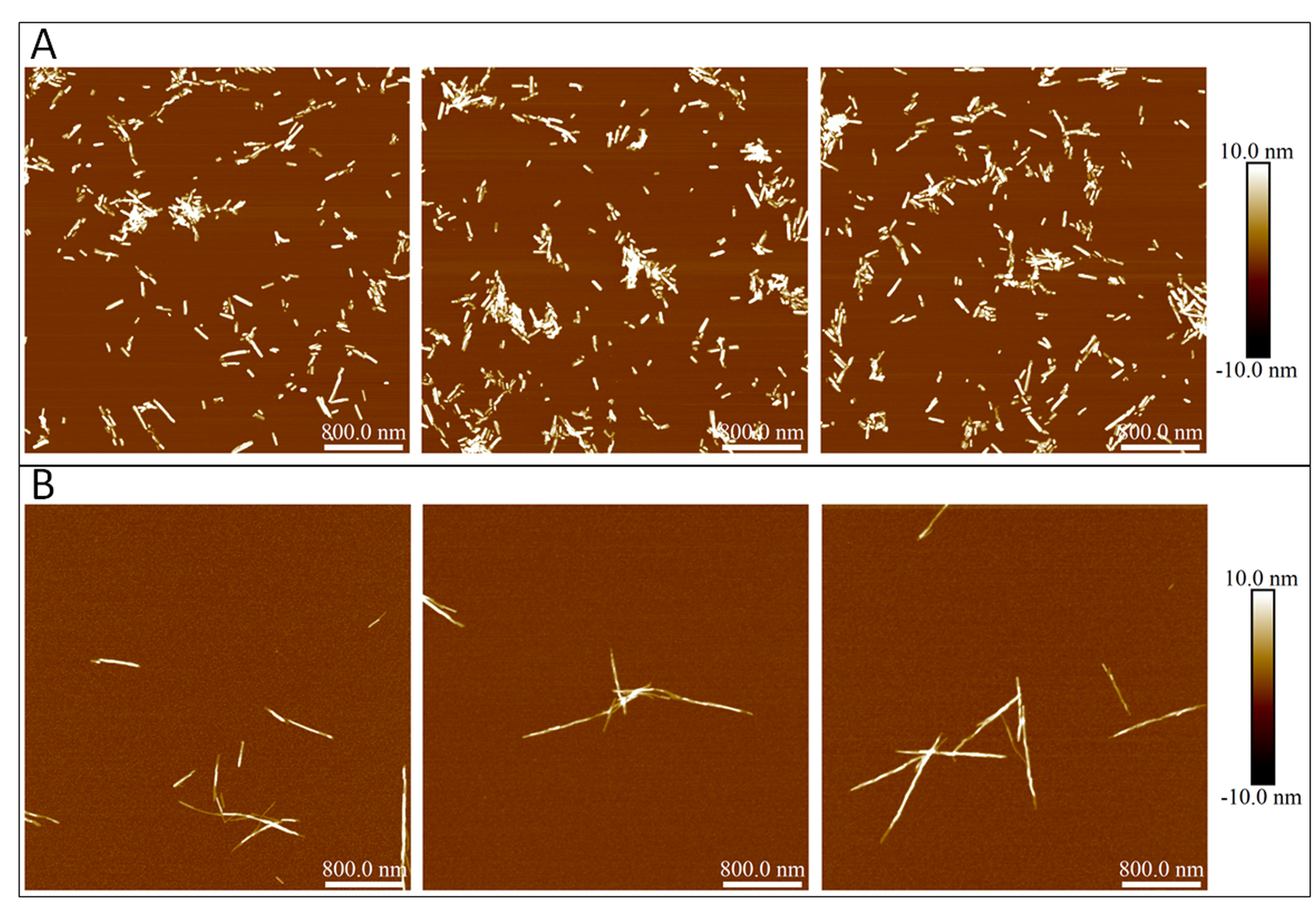

Supplement: Figure S3 [file peerj-07-7554-s003.png]

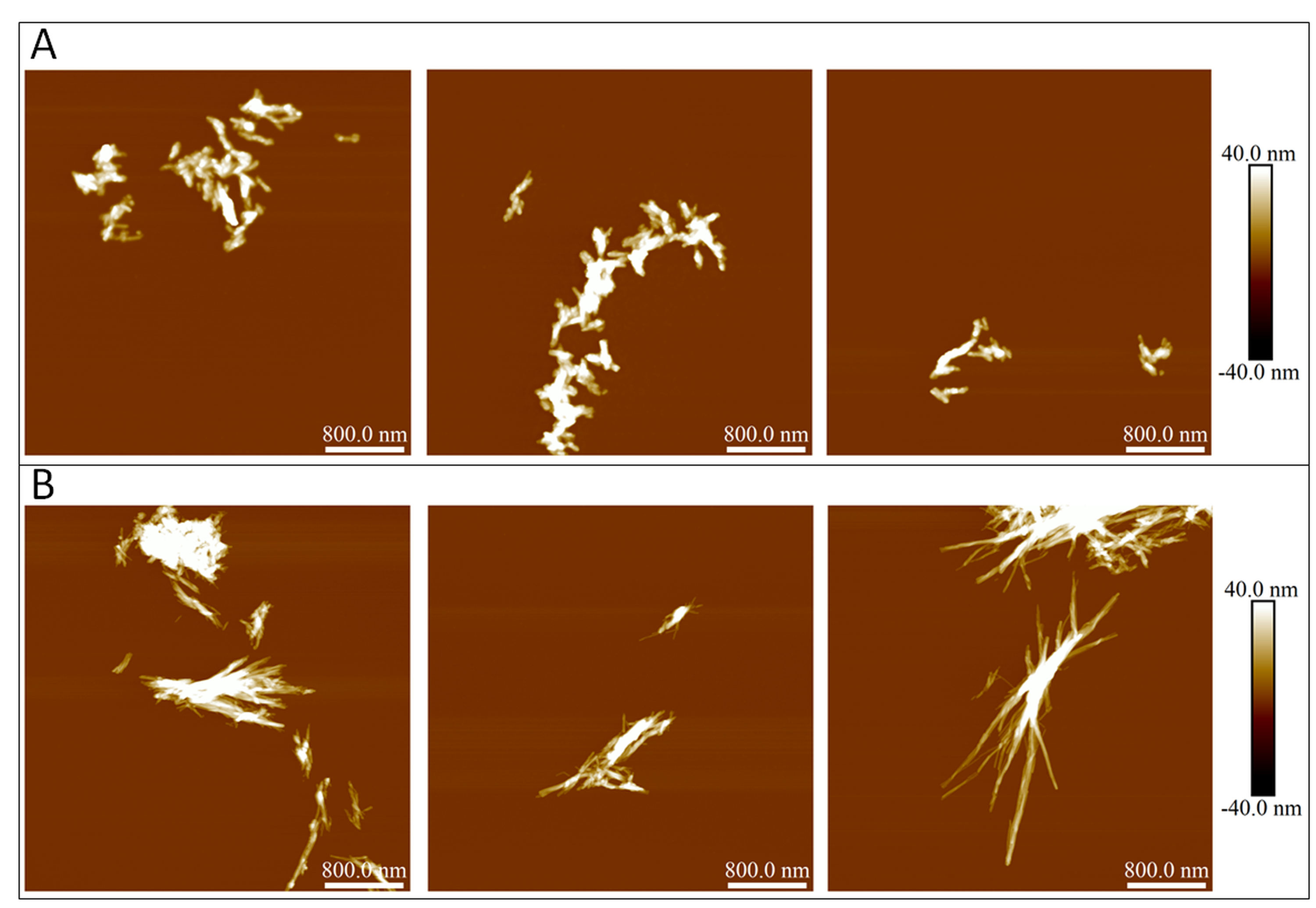

Supplement: Figure S4 [file peerj-07-7554-s004.png]

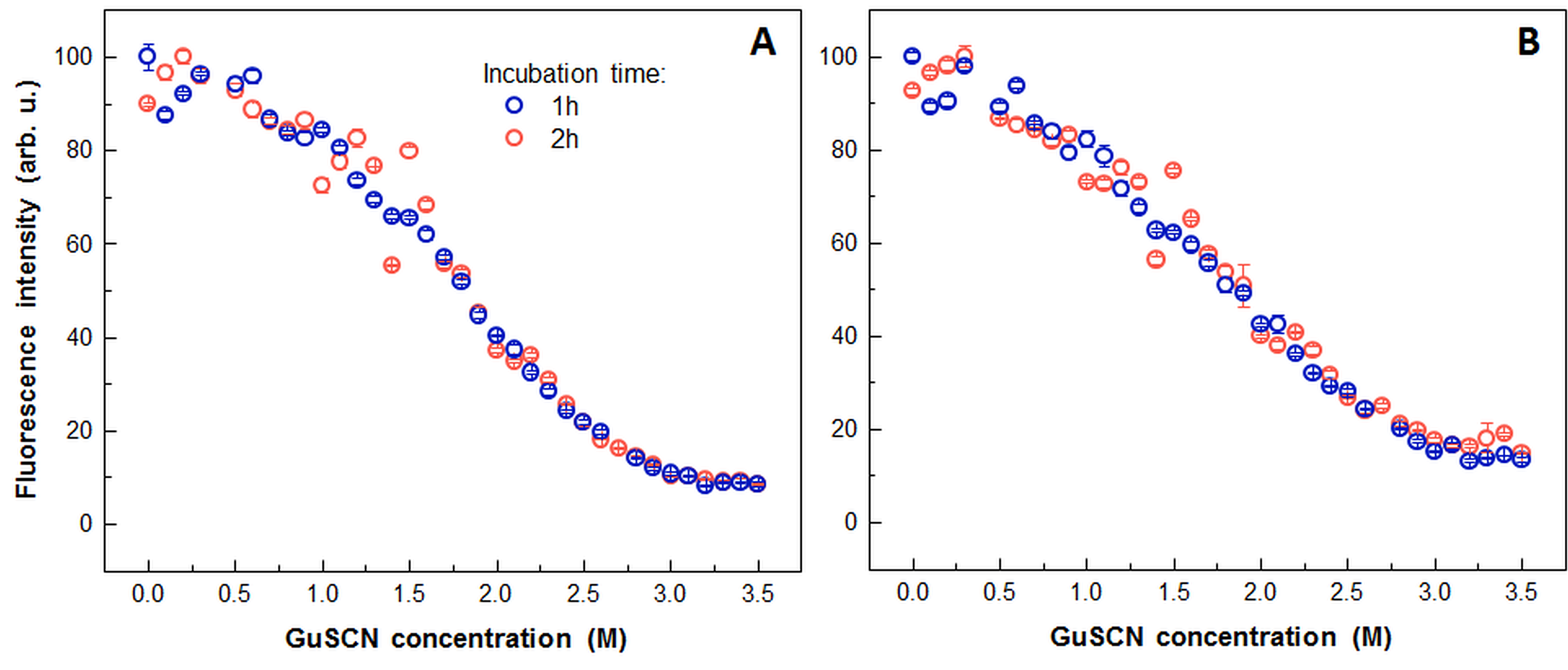

Supplement: Figure S5 [file peerj-07-7554-s005.png]

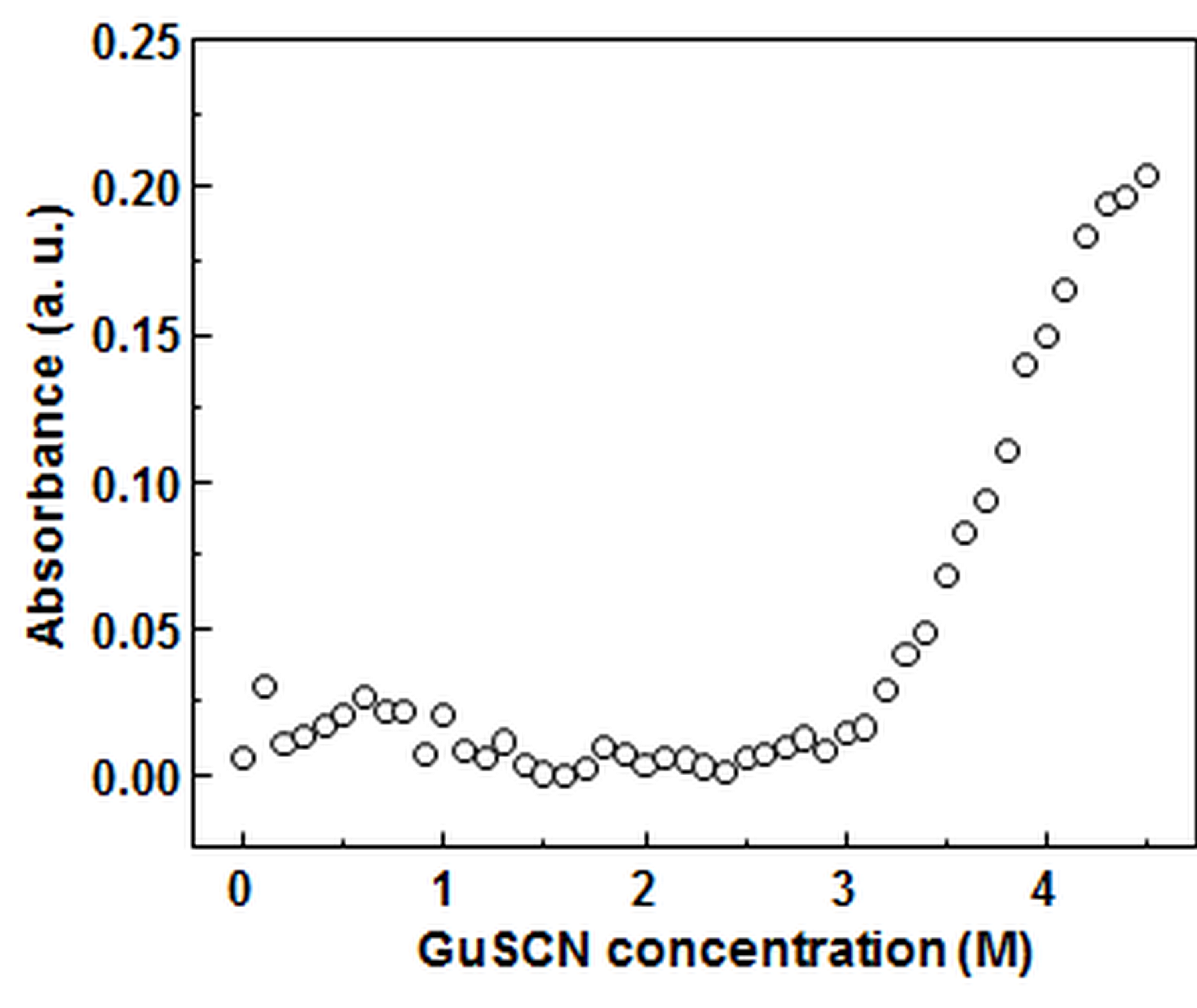

Supplement: Figure S6 — Fibril samples at different GuSCN concentrations were prepared as described in the dissociation assay method section, but at a higher volume (300 µL). The samples were incubated at 25 ° C for 1 hour and centrifuged for 20 min at 15,000 g and 4 ° C temperature. 150 uL of each sample’s supernatant was removed ant used for absorbance measurements. For each data point, the corresponding GuSCN buffer absorbance value was subtracted. [file peerj-07-7554-s006.png]

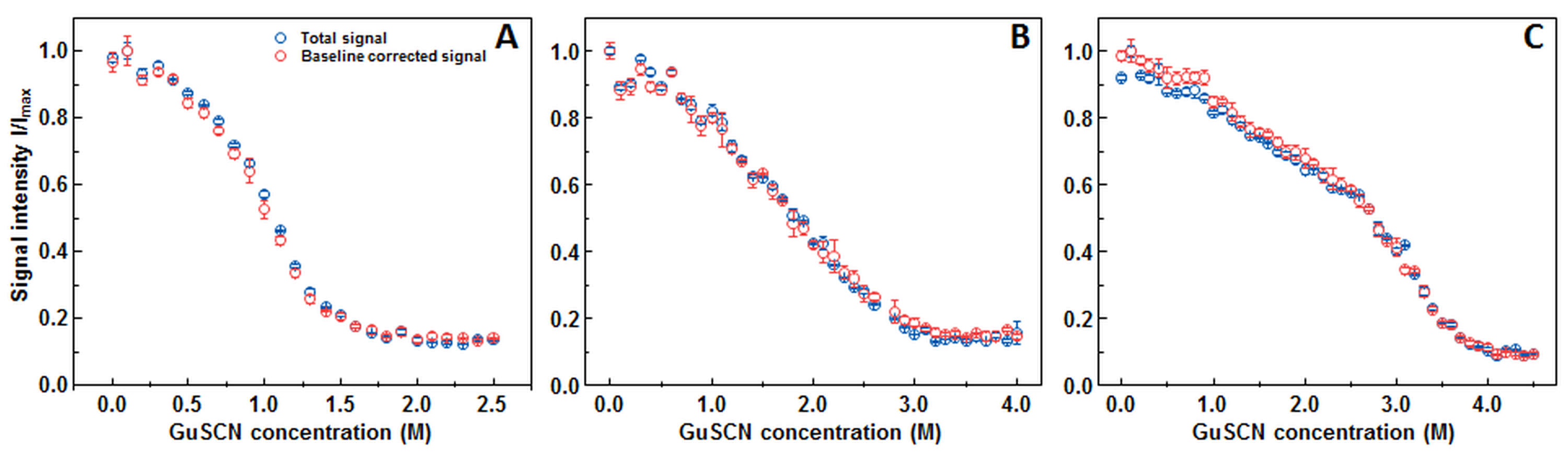

Supplement: Figure S7 — Insulin (A), prion protein (B) and lysozyme (C) fibril dissociation comparison when measuring by total signal intensity (blue) at 428 nm and when the signal is baseline corrected (red). Baseline was chosen as an average of signal intensities 14 nm from the 428 nm dbAF maxima peak (414 nm and 442 nm). [file peerj-07-7554-s007.png]
